# Supplementary material for: Geospatial mapping to assess the distribution and determinants of zero dose vaccination status hot spots among children in Ethiopia using EDHS 2019: Spatial and geographical weighted regression
Source: PLoS One. 2024 Dec 31;19(12):e0312610. doi: 10.1371/journal.pone.0312610 (PMC11687766; doi:10.1371/journal.pone.0312610)
Supplement: Attachment — Submitted filename: Point-by-point response to reviewers.pdf [file pone.0312610.s003.pdf]

Geospatial mapping to assess the distribution and determinants of zero dose vaccination status hot spots among children in Ethiopia using EDHS 2019: Spatial and geographical weighted regression

Muluken Chanie Agimas[1], Meron Asmamaw[1], Melese Kebede Hailu[2], Tigabu Kidie[1], Habtamu Wagnew Abuhay[1], Getaneh Awoke Yismaw[1], Nebiyu Mekonnen Derseh[1]

[1]Department of Epidemiology and Biostatistics, institute of public health, college of medicine and health scinece, university of Gondar, Gondar, Ethiopia.

[2]Department of emergency and critical care nursing, college of health sciences, Debre Tabor University, Debre Tabor, Ethiopia.

Authors address:

1. Muluken Chanie Agimas (MCA): mulukensrc12@gmail.com
2. Tigabu Kidie (TK): tigabukidie@gmail.com
3. Nebiyu Mekonnen Derseh (NMD): nebiyumek12@gmail.com
4. Meron Asmamaw (MA): merryalem101@gmail.com
5. Melese Kebede Hailu (MKH): melesk143@gmail.com
6. Habtamu Wagnew Abuhay (HWA): hab7wagnew@gmail.com
7. Getaneh Awoke Yismaw (GAY): getanehawoke9@gmail.com

Corresponding author:

(MCA): mulukensrc12@gmail.com

To: PLOS ONE

Submission ID: PONE-D-24-01734

Cover Letter for reviewer's and editor comments and authors responses

We would like to thank both the Editorial board members and reviewers for kind comments and suggestions. Accordingly, we have tried to correct and respond to those comments point by point, page by page, and line by line. Therefore, we prepared this author's response, the revised manuscript and the tracked changes of the manuscript. We accept most of your comments and some of your comments are needs justification to be clear for the scientific community.

Sincerely, with best regards!

Muluken Chanie Agimas

ID: https://orcid.org/0000-0003-0808-124X

Comments from review and editorial board	Authors response
**Editors comment**	**Authors response**
1. We note that Figures 2, 3, 4, 6, 7 and 8 in your submission contain [map/satellite] images which may be copyrighted. All PLOS content is published under the Creative	Thank you dear editors for you suggestion. Dear Editor, , the image for figures 2, 3, 4, 6, 7 and 8 was obtained from open source (a volunteer-driven open data platform). The link to access

Commons Attribution License (CC BY 4.0), which means that the manuscript, images, and Supporting Information files will be freely available online, and any third party is permitted to access, download, copy, distribute, and use these materials in any way, even commercially, with proper attribution. For these reasons, we cannot publish previously copyrighted maps or satellite images created using proprietary data, such as Google software (Google Maps, Street View, and Earth). For more information, see our copyright guidelines:

these shape files was first used by using the following link: africaopendata.org.

Then using this link the following interface is displayed

Then in the searching box we enter "Ethiopia" the following interface was displayed and the shape was downloaded in this site.

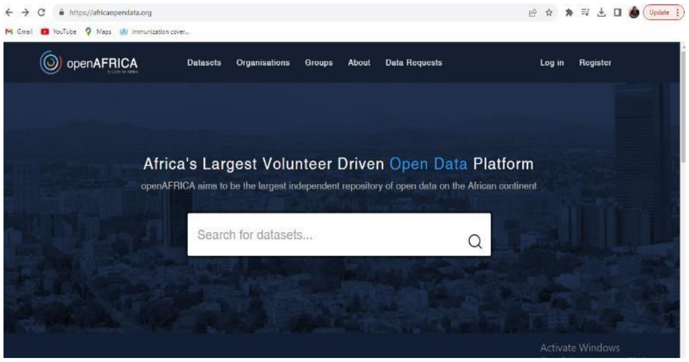

The final link for Ethiopia shape file is https://africaopendata.org/dataset/ethiopia-shapefiles

Then in the searching box we enter "Ethiopia" the following interface was displayed and the shape was downloaded in this site.

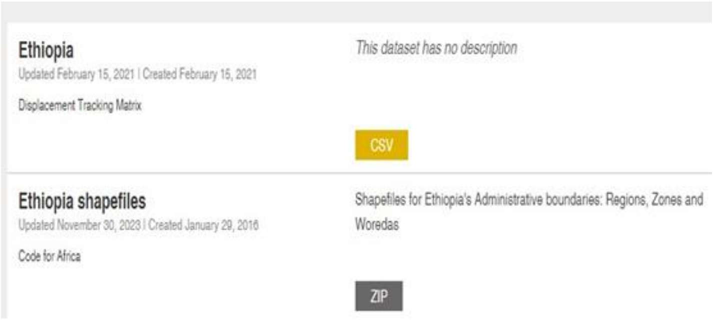

The final link for Ethiopia shape file is

https://africaopendata.org/dataset/ethiopia-shapefiles

2. You may seek permission from the original copyright holder of Figures 2, 3, 4, 6, 7 and 8 to publish the content specifically under the CC BY 4.0 license.	Dear Editor, definitely from DHS, we request the data for further spatial analysis. It is obvious that the spatial analysis is conducted using a shape file, and therefore, DHS International gives permission to use the data to analyze the data or generate an image using this data. To do this, the DHS International gives the coordinate file of Ethiopia. This coordinate file is important for mapping the data in the shape file. Generally, we request that DHS International use the data for spatial mapping using images. Accordingly, a permission letter was obtained from DHS International to approve the use of the data for mapping and creating the image. Mapping the data using a shape file or creating an image cannot disclose the confidentiality of the information to the participants. Because the location of the data was collected using a geographic positioning system (2 kilometers for urban clusters and 5 kilometers for rural clusters), That is why the DHS International gives permission for the mapping of the data using images. The permission letter said something like this: “The DHS Data may be used only for the purpose of statistical reporting and analysis, creating map images and only for your registered research. To use the data for another purpose, a new research project must be registered.
Reviewer one comments	Authors response

The introduction section lacks important information on national EPI vaccine coverages and estimates in the African countries.	Great view. Thank you so much for asking. We have made the necessary amendments.
The authors should review the definition of zero-dose status among children.<br>The authors should clarify their selection of children aged 12 to 35 months, given that numerous studies refer to children aged 12 to 23 months or 12 to 59 months, which would enhance comparability issues with other publications.	Thank you for your valuable comment. Dear Review, you asked a very important question. The reason for using the starting cutoff of 12 months is: first, to classify whether a child has zero dose, the child's age should be at least one year (12 months). Because the vaccine schedule is up to one year, it is impossible to interview those children aged less than 12 months (one year) because these children may have taken one of the vaccine doses up to one year. We have to select those children who are eligible to be interviewed for the question of whether they received zero-dose vaccines or not. For example, for a child under the age of 5 months, we cannot decide whether to give a zero dose or not. Because the child may take the vaccine at the age of 6, 7,... Up to the schedule end up (12 months). This cannot provide conclusive and valid evidence about zero-dose coverage. The reason we used the maximum cut-off of 35 months or did not include those women who had children aged more than 35 months was to reduce recall

	bias. The other reason why we use this cut-off is because previously, the EDHS 2019 was conducted and interviewed among women who had children aged 12-35 months. Because it is secondary data, we are forced to use the EDHS cutoff point.
The authors should consider expanding the discussion section with more studies conducted in the African region and recently published.	Dear reviewer, we have conducted a geographically weighted regression analysis. Which means the effect of each independent variable across the geographic regions, areas, or clusters. We face difficulty comparing the results with the previous findings because there was no spatial or geographical weighted regression previously. There are a lot of risk factor studies on zero-dose vaccination, but they are not used for spatial distribution comparison. For example, age may be a significant factor in cluster X, but it is hard to compare this finding with other previous findings. No similar study has reported the statistical significance of this factor in the same cluster so far. The only option was to just interpret this finding about why a particular predictor is significant in a given cluster. If we were to conduct a study on the risk factors for zero-dose vaccination status, there are a lot of studies on this area that we could easily compare

	with the previous studies. With this challenge and limitation, we try to elaborate and interpret the discussion in detail.
The authors should review the conclusion	Accepted and the amendment has made accordingly.
• The authors should review the manuscript for grammatical errors.	We have made grammatical revision of the whole manuscript.
While the introduction section clearly articulates the burden of zero-dose around the world, there is insufficient information about the status of the national Expanded Program on Immunization including the current coverage rates, and the progress and challenges, if any, It will be important to understand whether there are notable complex contextual issues that may contribute to the current status of the national EPI and zero-dose prevalence.	Dear reviewer thank you so much. This is an excellent view. We have found the trend of zero-dose children in Ethiopia, and we tried to add in the introduction section, including what are the factors or challenges to not achieving the target of vaccination coverage.

Furthermore, numerous research conducted in the African region (DRC, Nigeria, etc.) have been published in recent months/years. These studies have examined the prevalence of zero-dose and identified the elements that contribute to it. The findings of these studies can be effectively utilised to support the argument for this study.	Dear reviewer, we have conducted a geographically weighted regression analysis. Which means the effect of each independent variable across the geographic regions, areas, or clusters. We face difficulty comparing the results with the previous findings because there was no spatial or geographical weighted regression previously. There are a lot of risk factor studies on zero-dose vaccination, but they are not used for spatial distribution comparison. For example, age may be a significant factor in cluster X, but it is hard to compare this finding with other previous findings. No similar study has reported the statistical significance of this factor in the same cluster so far. The only option was to just interpret this finding about why a particular predictor is significant in a given cluster. If we were to conduct a study on the risk factors for zero-dose vaccination status, there are a lot of studies on this area that we could easily compare with the previous studies. With this challenge and limitation, we try to elaborate and interpret the discussion in detail.

Lines 23-24; 67<br>Zero-dose vaccine status refers to a child who has not yet received any childhood vaccines. The authors could check on this definition.<br>According to Gavi, Zero-dose children are those that have not received any ROUTINE vaccine. For operational purposes, Gavi defines zero-dose children as those who lack the first dose of diphtheria-tetanus-pertussis containing vaccine (DTP1).	. Dear reviewer, you are right. Different articles define zero-dose children in different ways, but in the EDHS 2019 or EDHS in general, the zero-dose child is defined as a child whose zero-dose vaccine status refers to a child who has not yet received any childhood vaccines. The report of EDHS was based on this definition, and thus we follow this definition. It is hard to revise this definition because we have already collected data. Secondly, our intention in this study is to assess the distribution of children who didn't receive any vaccine dose but not assess the distribution of the first dose of diphtheria, tetanus, and pertussis-containing vaccine (DTP1).
Line 25 “one child not access the vaccines” : one child did not access the vaccines	Accepted and revised accordingly.
Lines 25-26 “The efforts of identifying the factors of the zero-dose vaccine yet not addressed in Ethiopia”: The efforts to identify the factors contributing to the zero-dose vaccine have not yet been addressed in Ethiopia.	Accepted and revised accordingly.

Lines 34-35 To identify significant factors, a p-value <0.05 with 95% confidence was used.<br>Repetition. No need to present p-value and confidence level at the same time. They are complementary: “To identify significant factors, a p-value <0.05 was used” or “To identify significant factors, a 95% confidence was used”.	Accepted and revised accordingly.
Lines 36-37 The prevalence of zero-dose vaccination status among children aged 12-35 months old was 523 (16.3%, 95%CI, 15%–17.6%). The authors could clarify on this sentence, I don’t understand it. What is 523? Prevalence is a proportion.	Dear reviewer thank you so much for your comment. It was the number of children with zero doses. You are right; it creates confusion. So we have made a correction.
Line 36, 114 Question: Why age range of 12 to 35 months ?	Thank you for your valuable comment. Dear Review, you asked a very important question. The reason for using the starting cutoff of 12 months is: first, to classify whether a child has zero dose, the child's age should be at least one year (12 months). Because the vaccine schedule is up to one year, it is impossible to interview those children aged less than 12 months (one year) because these children may have taken one of the vaccine doses up to one year. We have to

	select those children who are eligible to be interviewed for the question of whether they received zero-dose vaccines or not. For example, for a child under the age of 5 months, we cannot decide whether to give a zero dose or not. Because the child may take the vaccine at the age of 6, 7,... Up to the schedule end up (12 months). This cannot provide conclusive and valid evidence about zero-dose coverage. The reason we used the maximum cut-off of 35 months or did not include those women who had children aged more than 35 months was to reduce recall bias. The other reason why we use this cut-off is because previously, the EDHS 2019 was conducted and interviewed among women who had children aged 12-35 months. Because it is secondary data, we are forced to use the EDHS cutoff point.
Line 41 Question: Home delivery of what ? please precise	It mean give birth at home and we revised accordingly.
Lines 47-48 As compared to the previous studies, the prevalence of zero-dose vaccination status was low in Ethiopia This conclusion is not correct, 16.3% is a high prevalence rate compared to average prevalence varying	Thank you so much, dear reviewer. We missed this article. Amendments have been made accordingly.

from 5.2% in upper-middle income countries to 11.1% in low-income countries, for a total pooled prevalence of 7.7%.	
Line 77-78 An increment of a zero-dose vaccine could be an indicator of not achieving the national vaccine coverage goal in sub-Saharan Africa (SSA) (90%) An increment of a zero-dose vaccine could be an indicator of not achieving the national vaccine coverage goal OVER TIME in sub-Saharan Africa (SSA) (90%)	Thank you so much, dear reviewer. We missed this article. Amendments have been made accordingly.
Line 84 Question: Who are zero-dose population? Are they different from zero-dose children? Please be consistent in the use terms.	Thank you. It mean zero dose children.
Lines 119 -120 The authors should distinguish independent variable related to household, to the woman and to the child	Dear reviewer, we have used the women-related factors and tried to clarify them accordingly. We face a challenge in including additional variables because it is secondary data, which misses many variables.

Lines 119 – 129; 125-127 While authors provided with the operational definition of “media exposure”, nothing was said regarding other independents variables.	Dear reviewer you are right; we define media exposure only. Other variables are too clear and don’t need additional clarification. Example. place of delivery, religion, age of the woman, ANC visit, etc. It doesn’t cause confusion about how it measures.
Lines 119-120: Religion is quoted twice	We accept it and revised accordingly.
Lines 208 - 209 zero dose vaccination status among children in Ethiopia was clustered (none randomly distributed)<br>Repetition. Clustered means not randomly distributed	Thank you for your view. We have accept the comment and revise accordingly.

The authors should consider expanding the discussion section with more studies conducted in the African region and recently published.<br>Lines 296 – 299 The authors compared the prevalence rate to those in Togo and Cameroon, but the three studies' definitions of zero-dose children and age ranges differed. The study in Togo defined zero-dose children as those who had not received the first dose of the diphtheria, tetanus, or pertussis vaccine, based on their immunization card, and their age ranged from 12 to 23 months. Similarly, the study in Cameroon defined zero-dose children as those who had not received any recommended vaccine antigen for their age, based on the Cameroon EPI calendar, and their age ranged from 0 to 24 months. The authors must take into account the comparability issues raised by the difference in their arguments.<br>Did the EDHS data rely on vaccination cards or maternal recall as the primary	Dear reviewer thank you so much for your comment. Dear reviewer, we try to add the studies done in Africa and Ethiopia to compare our findings. Regarding the age cutoff point, different articles set their own objectives. some studies may aim to understand the zero-dose children's coverage between the ages of 12 and 23, others may be 0–24, and so on. For this study, the reason for using the starting cutoff of 12 months is: first, to classify whether a child has zero dose, the child's age should be at least one year (12 months). Because the vaccine schedule is up to one year, it is impossible to interview those children aged less than 12 months (one year) because these children may have taken one of the vaccine doses up to one year. We have to select those children who are eligible to be interviewed for the question of whether they received zero-dose vaccines or not. For example, for a child under the age of 5 months, we cannot decide whether to give a zero dose or not. Because the child may take the vaccine at the age of 6, 7,... Up to the schedule end up (12 months). This cannot provide conclusive and valid evidence about zero-dose coverage. The reason we used the maximum cut-off of 35 months or

sources of information regarding childhood immunisation?	did not include those women who had children aged more than 35 months was to reduce recall bias. The other reason why we use this cut-off is because previously, the EDHS 2019 was conducted and interviewed among women who had children aged 12-35 months. Because it is secondary data, we are forced to use the EDHS cut of point.<br><br>The previous authors discussed their findings by considering the cut point of child age as a possible difference, challenge, or limitation. Otherwise, no study used the consistent cut point for child age. In this case, scholars recommended the following: considering as a limitation of the study, compared with the previous findings.<br><br>As much as possible, we tried to revise the discussion based on your recommendations.<br><br>Regarding the question<br><br>"Did the EDHS data rely on vaccination cards or maternal recall as the primary sources of

	information regarding childhood immunisation?” <br>The EDHS used the vaccination card and women's recall to provide information. If no card was available, it was based on maternal recall. That is why we used the cut-off point of women having children aged 35 months, not up to 59 months, to reduce the recall bias.
The authors should clarify the primary source used for this study. Lines 346 – 347 16% is a high prevalence not low. The pooled prevalence rate of zero-dose children is 7.7% in LMIC, with an average of 11.1% in Low-income countries (Cata-Preta BO, 2021) in a systematic review	Dear reviewer the findings of the current study are not consistently in line with or higher or lower than the previous findings. It is lower than some findings, higher than some studies conducted previously, and in line with some previous studies. Which makes it hard to interpret or compare with the previous findings. In this case, it is better to compare with the target.

including 92 countries. The studies referred to here with high prevalence rates have a very specific context that has been explained, and they cannot be considered flagship studies for the prevalence of zero-dose children.	The target vaccination is to reach 90%, or reduce the uptake of vaccine coverage by less than 10%. But our findings report 16.3%. Which is still not achieving the target.
**Reviewer two comments**	**Authors response**
"The article is very interesting, it presents a scientific and methodological rigor, with adequate statistical analysis to respond to the main objective of the study.<br>I suggest some corrections so that the text achieves the excellence required in this journal:<br>The abstract has many acronyms.	Dear reviewer thank you so much for your scientific contribution and valuable comments. Based on your recommendation, we have made revisions to the abstract section.
The introduction is based on an updated review of the scientific literature. The methodology is well explained. I only suggest briefly explaining what the EDHS2019 study is and how it accounted for the ANC visit variable.	Dear reviewer, thank you, and we have made a revision accordingly.

The results are well presented but I suggest some corrections:Table 2 shows the 95% confidence interval of the RR, open tables 3 and 4 on the sides, and have homogeneity of decimal places in all tables.	Dear reviewer, thank you so much for the necessary amendments you have made.
The Discussion is backing up the results.	Dear reviewer, we have conducted a geographically weighted regression analysis. Which means the effect of each independent variable across the geographic regions, areas, or clusters. We face difficulty comparing the results with the previous findings because there was no spatial or geographical weighted regression previously. There are a lot of risk factor studies on zero-dose vaccination, but they are not used for spatial distribution comparison. For example, age may be a significant factor in cluster X, but it is hard to compare this finding with other previous findings. No similar study has reported the statistical significance of this factor in the same cluster so far. The only option was to just interpret this finding about why a particular predictor is significant in a given cluster. If we were to conduct a study on the risk factors for zero-dose vaccination status, there are a lot of studies on this area that we could easily compare

	with the previous studies. With this challenge and limitation, we try to elaborate and interpret the discussion in detail.

Thank you so much for your very impressive scientific contribution.
