# Supplementary material for: Geospatial mapping to assess the distribution and determinants of zero dose vaccination status hot spots among children in Ethiopia using EDHS 2019: Spatial and geographical weighted regression
Source: PLoS One. 2024 Dec 31;19(12):e0312610. doi: 10.1371/journal.pone.0312610 (PMC11687766; doi:10.1371/journal.pone.0312610)
Supplement: Attachment — Submitted filename: Point-by-point response to reviewers.pdf [file pone.0312610.s004.pdf]

Geospatial mapping to assess the distribution and determinants of zero dose vaccination status hot spots among children in Ethiopia using EDHS 2019: Spatial and geographical weighted regression

Muluken Chanie Agimas[1], Meron Asmamaw[1], Melese Kebede Hailu[2], Tigabu Kidie[1], Habtamu Wagnew Abuhay[1], Getaneh Awoke Yismaw[1], Nebiyu Mekonnen Derseh[1]

[1]Department of Epidemiology and Biostatistics, institute of public health, college of medicine and health scinece, university of Gondar, Gondar, Ethiopia.

[2]Department of emergency and critical care nursing, college of health sciences, Debre Tabor University, Debre Tabor, Ethiopia.

Authors address:

1. Muluken Chanie Agimas (MCA): mulukensrc12@gmail.com
2. Tigabu Kidie (TK): tigabukidie@gmail.com
3. Nebiyu Mekonnen Derseh (NMD): nebiyumek12@gmail.com
4. Meron Asmamaw (MA): merryalem101@gmail.com
5. Melese Kebede Hailu (MKH): melesk143@gmail.com
6. Habtamu Wagnew Abuhay (HWA): hab7wagnew@gmail.com
7. Getaneh Awoke Yismaw (GAY): getanehawoke9@gmail.com

Corresponding author:

(MCA): mulukensrc12@gmail.com

To: PLOS ONE

Submission ID: PONE-D-24-01734

Cover letter for second round reviewer's comment and authors responses

We would like to thank both the Editorial board members and reviewers for kind comments and suggestions. Accordingly, we have tried to correct and respond to those comments point by point, page by page, and line by line. Therefore, we prepared this author's response, the revised manuscript and the tracked changes of the manuscript. We accept most of your comments and some of your comments are needs justification to be clear for the scientific community.

Sincerely, with best regards!

Muluken Chanie Agimas

ID: https://orcid.org/0000-0003-0808-124X

**Comments from reviewer**	**Authors response**
**Reviewer one comment**	**Authors response**
Data availability statement: The authors did not provide a link to access the data or specify how to do so.	Dear reviewer, it is very interesting question. Dear reviewer as DHS international restriction, the raw data cannot be shared. Sharing this raw data violates the DHS international data use guideline. They release the data to us only for the analysis purpose not for online submission and publication. The raw data can be accessed online at http://www.dhsprogram.com But all data output is in the manuscript. Thus, we tried to

	amend the data availability statement accordingly.
Line 151-152 “A two-stage cluster sampling method was used to select 3208 women (participants) who had children aged 12-35 months in the EAs” and Line 209 “Of the total 3028 women who had children aged 12-35 months old” : the author should fix the real sample size.	Sorry for inconsistency. The real sample size was 3028. So. We accepted and the amendment has made accordingly.
Line 217 : “The prevalence of zero-dose vaccination status among children aged 12-35 months old was 523” : prevalence is a proportion; the authors should add the denominator.	Dear reviewer amendment has made accordingly. It was because of editorial error.
Line 310 – 312 : The current study does not have the same age range as the referral study (1), so the author should be cautious when interpreting the findings. What effect might a narrow age range have on the current research's prevalence when compared to a study with a wider age range?	Accepted and amendment has made accordingly.

Line 315-317 What is lower here ? your prevalence or the one conducted between 2010 and 2020. Please clarify.	Dear reviewer thank you so much. This is an excellent view. We accepted your comment and revision has made accordingly. Regarding the term lower, it mean that the zero dose child in this study is lower than a study conducted between 2010 and 2020 using demographic health survey data set in sub-Saharan Africa (9.1%).
Line 366 “the lack of comparable studies makes it more difficult to compare with the previous findings” : ‘The lack of studies with the same age range, makes it more difficult to compare the results to earlier findings’ this sounds more specific.	Thank you so much dear reviewer. We accept the comment and revision has made accordingly.
Line 46 and 368 “Ethiopia did not achieve the target of vaccination coverage” : Is this study intended to evaluate vaccine coverage? Which target of vaccine coverage are you talking about here? Did you calculate the vaccine coverage in this study? For which antigen? The research findings, in my opinion, do not support this statement.	Dear reviewer, sorry for the confused word. It mean the prevalence of zero dose vaccination was low. So amendment has made accordingly.

Thank you so much for your very impressive scientific contribution.
